# Comparison of Clinical Outcomes Between Culture-Positive and Culture-Negative Sepsis and Septic Shock Patients: A Meta-Analysis

**DOI:** 10.7759/cureus.35416

**Published:** 2023-02-24

**Authors:** Muhammad S Afzal, Raj Nandan Chennuri, Husnain Naveed, Bansari Raveena Bai, Rutaba Hanif, Zoha Shahzad, Muhammed Umer, Faraz Saleem

**Affiliations:** 1 Department of Medicine, Carle Foundation Hospital, Urbana, USA; 2 Department of Medicine, Louisiana State University Shreveport, Shreveport, USA; 3 Department of Internal Medicine, Andhra Medical College, Visakhapatnam, IND; 4 Department of Internal Medicine, Shifa Tameer-e-Millat University, Shifa College of Medicine, Islamabad, PAK; 5 Department of Internal Medicine, Peoples University of Medical and Health Sciences for Women (PUMHSW), Nawabshah, PAK; 6 Department of Internal Medicine, Karachi Medical and Dental College, Karachi, PAK; 7 Department of Internal Medicine, Fatima Jinnah Medical University, Lahore, PAK; 8 Department of Internal Medicine, Dow University of Health Sciences (DUHS) Civil Hospital Karachi, Karachi, PAK; 9 Department of Internal Medicine, Akhtar Saeed Medical and Dental College, Lahore, PAK

**Keywords:** meta-analysis, culture negative, culture positive, septic shock, sepsis

## Abstract

This meta-analysis has been conducted to compare the clinical outcomes between culture-positive and culture-negative sepsis or septic patients. The present meta-analysis is reported as per the Preferred Reporting Items for Systematic Reviews and Meta-Analyses (PRISMA) guidelines. Electronic databases, including PubMed and EMBASE, were searched by two authors independently from the inception to January 25, 2023, using the following key terms: “culture positive,” “culture negative,” “sepsis,” and “septic shock.” The primary outcome assessed in the present meta-analysis was all-cause mortality. Secondary outcomes included the need for mechanical ventilation, renal replacement therapy, length of ICU stay in days, and length of hospital stay in days. Total 10 studies met the inclusion criteria and were included in the meta-analysis involving 23,973 patients. No statistically significant difference was found between culture-positive and culture-negative patients in terms of all-cause mortality (risk ratio [RR]: 1.09, 95% CI: 0.95-1.24, p-value: 0.23), the need for mechanical ventilation (RR: 0.99, 95% CI: 0.93-1.05, p-value: 0.79), renal replacement therapy requirements (RR: 1.11, 95% CI: 0.95-1.31, p-value: 0.19), and ICU length of stay (mean difference [MD]: 1.70 days, 95% CI: -1.10, 4.49, p-value: 0.23). However, The mean hospital length of stay in days was significantly longer in patients in a culture-positive group compared to the culture-negative group (MD: 3.04, 95% CI: 2.25-3.82, p-value<0.001). In conclusion, the present meta-analysis of 10 studies, including 23,973 patients, found no significant differences in all-cause mortality, need for mechanical ventilation, need for renal replacement therapy, and length of ICU stay between culture-positive and culture-negative sepsis or septic patients. However, a significant difference was found in hospital length of stay, with culture-positive patients having a longer stay.

## Introduction and background

Sepsis is defined as organ dysfunction caused by a dysregulated host response to infection and is a leading cause of mortality among critically ill patients [1]. The incidence of septicemia is expected to increase due to the aging population and the presence of multiple comorbidities. However, the fatality rate has gradually decreased with the implementation of resuscitation protocols and bundled care [2]. The treatment guidelines for sepsis and septic shock have undergone revisions and updates over the past 20 years. Bacteria are known to be the major cause of sepsis pathogens, and early selection of appropriate antibiotics can improve survival chances [3]. The widely accepted approach is to use broad-spectrum antibiotics, and research has shown that prompt administration of antibiotics is crucial in reducing the risk of death for patients [4]. A blood culture test can help adjust empirical antibiotic treatment appropriately [5]. A positive blood culture result is often considered an indicator of a high bacterial load and is associated with worse patient outcomes [6]. Administering antibiotics promptly and correctly can lower the risk of death. However, determining the appropriate antibiotics in the early stages of sepsis can be difficult, as a significant proportion of patients, ranging from one-third to two-thirds, have microorganisms that cannot be grown in culture, which increases their risk of death [7]. Sepsis encompasses a wide range of microorganisms, only a fraction of which are microbiologically documented. Sepsis or septic shock without microbiologically documented infection is known as culture-negative sepsis or septic shock [8]. Diagnosing culture-negative septic shock involves identifying septic shock without finding positive evidence of pathogens in blood, sputum, bodily fluids, or tissues. Previous studies have reported that culture-negative cases comprise 28-49% of all sepsis patients [9-10]. Septic shock is a heterogeneous syndrome that can impact different vital organs in varying ways. A recent study found that a quicker detection time of cultures was related to the severity of the disease but not to the risk of death in patients with septic shock [11]. Whether patients with culture-positive sepsis or septic shock behave differently from those with culture-negative sepsis or septic shock remains controversial based on currently available literature [7, 11-13]. Since the publication of a previous systematic review and meta-analysis conducted by Li Y et al. [8], three new studies have been conducted. These studies need to be incorporated into the current understanding of the subject. A meta-analysis provides an opportunity to incorporate these new findings and to provide an updated synthesis of the existing evidence base. The present meta-analysis has been conducted to compare the clinical outcomes between culture-positive and culture-negative sepsis or septic patients.

## Review

Methodology

The present meta-analysis is reported as per the Preferred Reporting Items for Systematic Reviews and Meta-Analyses (PRISMA) guidelines.

Search Strategy and Study Selection

Electronic databases, including PubMed and EMBASE, were searched by two authors independently from the inception to January 25, 2023, using the following key terms: “culture positive,” “culture negative,” “sepsis,” and “septic shock.” The key terms were joined using Boolean algebra while performing the search. The reference list of each included study was also manually searched. All records were imported to EndNote X9. After removing duplicates, the initial screening of articles was done using the title and abstract. Full texts of all eligible articles were retrieved and screened for pre-defined inclusion and exclusion criteria. Articles met the inclusion and exclusion criteria included in the present meta-analysis. Any disagreement between two authors was resolved via consensus or discussion with a third author if needed.

Eligibility Criteria

All studies included that fulfilled the following criteria: (a) observational studies including prospective and retrospective cohort and case-control studies; (b) patients with sepsis and septic shock; (c) studies reported any of the outcomes assessed in the present meta-analysis; (d) comparing culture-positive and culture-negative patients. We excluded reviews, editorials, case reports, and case series.

Outcomes Measured

The primary outcome assessed in the present meta-analysis was all-cause mortality. Secondary outcomes included the need for mechanical ventilation, the need for renal replacement therapy, the length of ICU stay in days, and the length of hospital stay in days.

Data extraction and Data Synthesis

Data were extracted from the included studies using a data collection form developed on Microsoft Excel. Data were extracted, including the first author's name, year of publication, groups, sample size, and outcomes. Data were extracted by one author, and the second author cross-checked it and entered it into RevMan software for data analysis. Statistical analyses were performed using Review Manager Version 5.4.1 (RevMan, The Cochrane Collaboration, Oxford, UK). We calculated the risk ratio (OR) along with 95% CI to compare categorical outcomes between culture-positive and culture-negative patients. For continuous outcomes, we used mean difference (MD) with their 95% CI. A p-value < 0.05 was set as the threshold of statistical significance. Heterogeneity was assessed using I-square. For the I-square value of >50%, random effect mode was used, otherwise fixed-effect model was used.

Results

**Figure 1:**
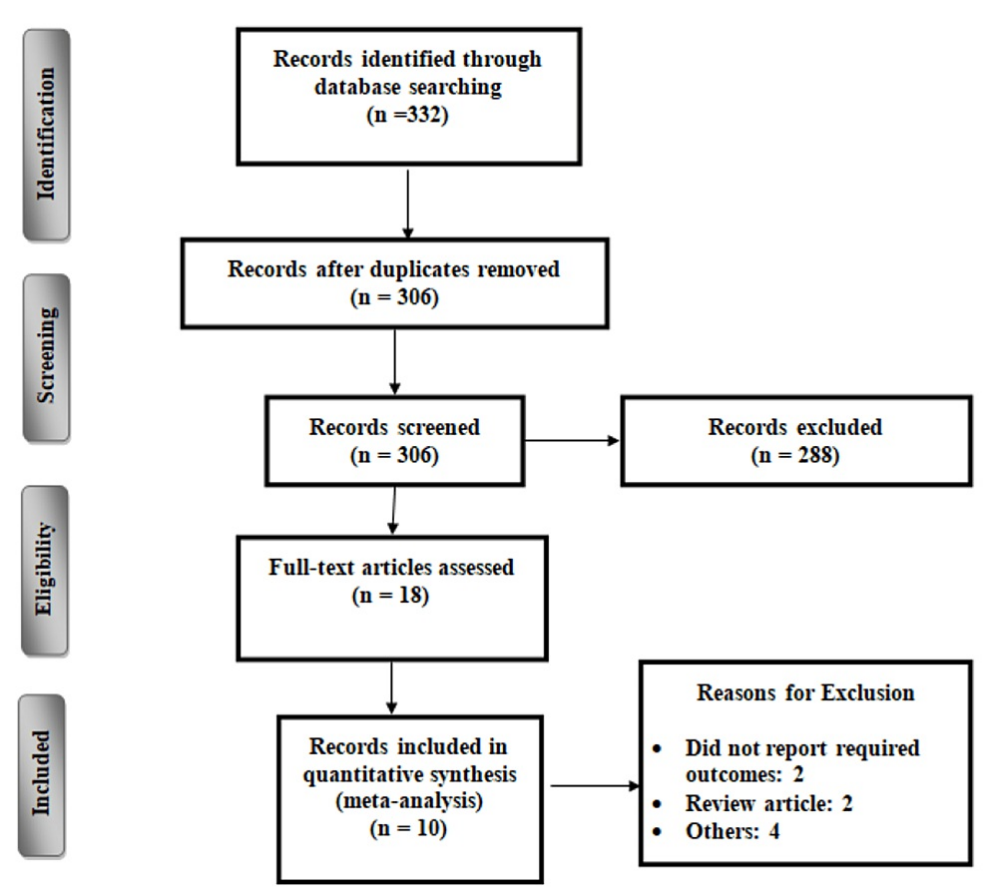
PRISMA flowchart of selection of studies. PRISMA: Preferred Reporting Items for Systematic Reviews and Meta-Analyses.

Figure 1 shows the process of study selection. The overall search yielded 332 studies. After removing duplicates, 306 studies were initially screened using their titles and abstracts. Full texts of 18 studies were retrieved and assessed for eligibility criteria. Overall, 10 studies met the inclusion criteria [6-7, 11-18] and were included in the meta-analysis involving 23,973 patients. The characteristics of the included studies are shown in Table 1. The majority of the included studies were retrospective cohorts [7, 11-14, 16-18]. 

**Table 1:** Characteristics of included studies.

Author	Year	Study Design	Groups	Sample Size
Bast J et al. [14]	2015	Retrsospective cohort	Culture positive	296
			Culture negative	288
Chua CB et al. [15]	2022	Case-control	Culture positive	311
			Culture negative	177
Easaw SM et al. [16]	2017	Retrsospective cohort	Culture positive	38
			Culture negative	42
Hazwani TR et al. [17]	2020	Retrsospective cohort	Culture positive	30
			Culture negative	179
Huang H et al. [18]	2022	Retrsospective cohort	Culture positive	114
			Culture negative	124
Kethireddy S et al. [12]	2018	Retrsospective cohort	Culture positive	6019
			Culture negative	2651
Kim JS et al. [11]	2021	Retrsospective cohort	Culture positive	1012
			Culture negative	706
Phua J et al. [6]	2013	Prospective cohort	Culture positive	586
			Culture negative	415
Sigakis MJ et al. [13]	2020	Retrsospective cohort	Culture positive	1105
			Culture negative	9288
Yang SC et al. [7]	2021	Retrsospective cohort	Culture positive	274
			Culture negative	318

All-cause Mortality

**Figure 2:**
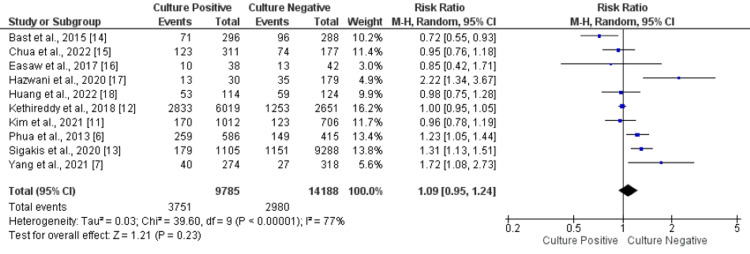
Forest plot comparing incidence of all-cause mortality. Sources: References [6-7, 11-18]

A total of 10 studies, including 23,973 patients, were included, and the all-cause mortality was about 38.3% in the culture-positive group and 21.0% in the culture-negative group. No statistically significant difference was found in all-cause mortality between the two study groups (RR: 1.09, 95% CI: 0.95-1.24, p-value: 0.23), as shown in Figure 2. Significant heterogeneity was found among the study results (I-square: 77%, p-value: 0.001).

Need of Mechanical Ventilation

**Figure 3:**
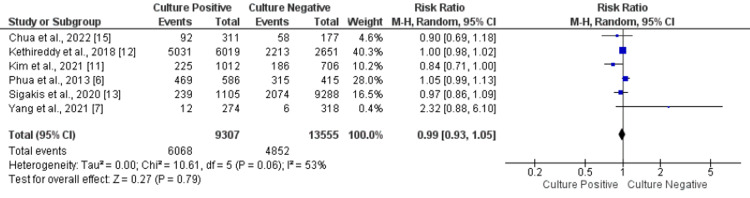
Forest plot comparing the need of mechanical ventilation between the two groups. Sources: References [6-7, 11-13, 15]

Six of the included compared the need for mechanical ventilation in patients in the culture-positive and culture-negative groups. The need for mechanical ventilation was about 47.76% (6068/9307 in the culture-positive group and 4852/13555 in the culture-negative group). No statistical difference was found in need for mechanical ventilation between the two groups (RR: 0.99, 95% CI: 0.93-1.05, p-value: 0.79), as shown in Figure 3. Significant heterogeneity was found among the study results (I-square: 53%, p-value: 0.79).

Need of Renal Replacement Therapy

**Figure 4:**
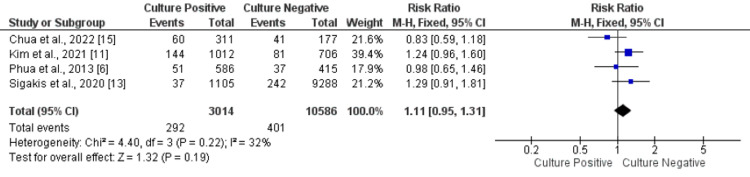
Forest plot comparing need of renal replacement therapy between the two groups. Sources: References [6, 11, 13, 15]

Four studies were included in the present meta-analysis to compare the renal replacement therapy requirements between patients in the culture-positive and negative groups. There was no statistically significant difference in the renal replacement therapy requirements between the two study groups (RR: 1.11, 95% CI: 0.95-1.31, p-value: 0.19), as shown in Figure 4. No significant heterogeneity was found among the study results (I-square: 32%, p-value: 0.22).

ICU Length of Stay

**Figure 5:**
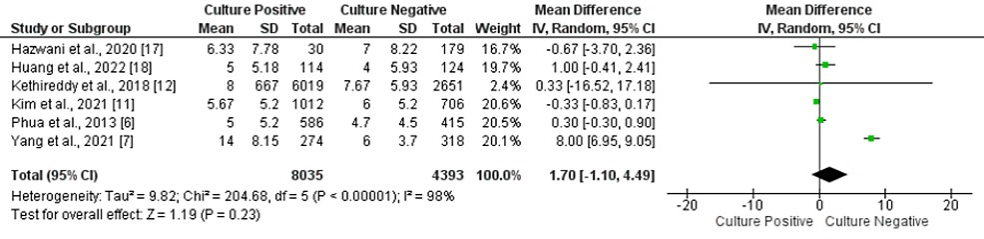
Forest plot comparing length of ICU stay between the two groups. Sources: References [6-7, 11-12, 17-18]

Six of included studies compared the length of stay between two groups. No significant differences were there between the two groups in overall ICU length of stay (MD: 1.70 days, 95% CI: -1.10, 4.49, p-value: 0.23), as shown in Figure 5. Significant heterogeneity was found among the study results (I-square: 98%, p-value=0.001).

Hospital Length of Stay

**Figure 6:**
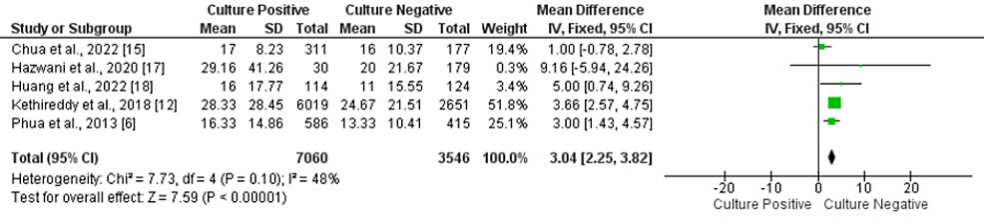
Forest plot comparing length of hospital stay between the two groups. Sources: References [6, 12, 15, 17-18]

Five studies were included in the present meta-analysis to assess the hospital length of stay in days. The mean hospital length of stay in days was significantly longer in patients in the culture-positive group compared to the culture-negative group (MD: 3.04, 95% CI: 2.25-3.82, p-value<0.001), as shown in Figure 6. No significant heterogeneity was found among the study results (I-square: 48%, p-value: 0.1).

Discussion

The present meta-analysis of seven studies, including 23,973 patients, showed that no significant difference was found between culture-positive and culture-negative sepsis or septic patients in all-cause mortality, the need for renal replacement therapy, the need for mechanical ventilation, and length of stay in the ICU. However, the overall length of hospital stay was significantly shorter in patients with culture-negative sepsis or sepsis compared to their counterparts. The meta-analysis conducted in the past also found that no significant difference is there between culture-positive and culture-negative sepsis and septic shock patients in terms of risk of mortality, the need for mechanical ventilation, renal replacement therapy, and length of stay in the ICU [8]. A crucial question arises as to why some patients presenting with sepsis or septic shock symptoms have a positive culture for an infection. Possible explanations for culture-negative sepsis or septic shock are multifaceted. For example, small amounts of bacteria or the temporary presence of bacteria in the bloodstream can lead to infections that cannot be cultured in these patients. Additionally, the growing practice of prescribing antibiotics before conducting a culture test when patients receive outpatient care has been found to independently contribute to culture negativity in sepsis patients [13, 19]. Another possibility is the increasing prevalence of non-bacterial sepsis, such as viral sepsis, in which conventional methods have limited capability in detecting pathogens [20]. Lastly, non-infectious illnesses like metabolic or inflammatory disorders may be misdiagnosed and attributed to septic conditions [21]. The current meta-analysis found that the length of hospital stay was longer in culture-positive patients compared to culture-negative patients. Since septic shock and sepsis are heterogeneous syndromes, the infection sites are also quite different between the two groups. A retrospective study conducted by Labelle AJ et al. showed that culture-positive patients with lung and intra-abdominal infections were associated with poor clinical outcomes [22]. A negative culture may result in a greater likelihood of responding to initial antibiotic treatments, leading to a milder form of the disease. Clinical outcomes may be linked not only to the source of infection but also to the management of sepsis and septic shock [8]. According to the Surviving Sepsis Campaign guidelines, administering broad-spectrum antibiotics as soon as possible is recommended to improve outcomes in both culture-negative and culture-positive sepsis cases. The guidelines state that each hour of delay in starting effective antibiotics from the onset of the septic shock increases mortality risk [4, 23]. The guidelines also suggest that early initiation of antimicrobial therapy is considered appropriate for culture-negative septic patients if they present with symptoms that match national guidelines for the specific clinical syndrome. Multiplex PCR amplification techniques are recommended to accurately quantify the presence of fungi, viruses, and bacteria to determine the true-negative and false-negative rates of cultures [24].

Study Limitations

The present meta-analysis has certain limitations. Firstly, many outcomes, such as the length of hospital stay, length of ICU, the need for renal replacement therapy, and the need for mechanical ventilation, were not analyzed in most of the studies included in the meta-analysis. Secondly, the suspected source of infection might be an important effect modifier, as certain infection sources have lower mortality rates and higher rates of culture positivity, and vice versa. However, not every study included in the meta-analysis specified the suspected source of infection.

## Conclusions

In conclusion, the present meta-analysis of 10 studies, including 23,973 patients, found no significant differences in all-cause mortality, need for mechanical ventilation, need for renal replacement therapy, and length of ICU stay between culture-positive and culture-negative sepsis or septic patients. However, a significant difference was found in hospital length of stay, with culture-positive patients having a longer stay. However, only some studies assessed the included outcome; future studies are required with a larger sample size to assess the outcomes differences between patients with positive-culture and negative-culture sepsis and septic shock.
